# Repeated-sprint sets test: a new method for evaluating and forecasting fitness in elite young male soccer players

**DOI:** 10.1038/s41598-024-58974-z

**Published:** 2024-04-12

**Authors:** Mohamed Amin Selmi, Halil ibrahim Ceylan, Raouf Hammami, Radhouane Haj Sassi, Francisco Tomás González-Fernández, Ryland Morgans, Nicola Luigi Bragazzi

**Affiliations:** 1 Higher Institute of Sport and Physical Education of Ksar-Said, Manouba University, Tunis, Tunisia; 2 Tunisian Research Laboratory ‘Sports Performance Optimization’, National Center of Medicine and Science in Sports (CNMSS), Tunis, Tunisia; 3 Physical Education and Sports Teaching Department, Kazim Karabekir Faculty of Education, Ataturk University, 25240 Erzurum, Turkey; 4 Tunisian Research Laboratory ‘Sports Performance Optimization’, National Center of Medicine and Science in Sports (CNMSS), Tunis, Tunisia; 5 Department of Physical Education and Sports, Faculty of Sport Sciences, University of Granada, 18071 Granada, Spain; 6 School of Sport and Health Sciences, Cardiff Metropolitan University, Cardiff, UK; 7 Laboratory for Industrial and Applied Mathematics (LIAM), Department of Mathematics and Statistics, York University, Toronto, Canada; 8 Human Nutrition Unit (HNU), Department of Food and Drugs, Medical School, University of Parma, Building C, Via Volturno, 39, 43125 Parma, Italy

**Keywords:** Repeated sprint sets, Physical fitness, Youth, Soccer, Physiology, Risk factors

## Abstract

The objective of the current study was to explore the correlation between repeated sprint sets (RSS) ability and several physical attributes, including maximum sprint speed, maximal aerobic speed, maximal anaerobic speed, aerobic capacity, and explosive strength. Moreover, the aim was to assess the suitability of RSS as a comprehensive evaluation tool for physical qualities and to determine which physical field tests most accurately predict RSS in elite young male soccer players. A total of thirty-two young elite male soccer players (mean age 14.6 ± 0.3 years; predicted years from peak height velocity (PHV): − 0.4 ± 0.3; years in training: 3.7 ± 0.5) voluntarily participated in the study. The players participated in eight consecutive specific physical tests, with a minimum 72-h recovery between each session to minimize the impact of fatigue during the second trial. The participants completed the tests in the following order: RSS test, Vam-Eval test, a constant velocity test performed until exhaustion at 100% of vVO_2_max (tlim100), 20-m Multi-Stage Shuttle Run test (V_MSRT_), Yo-Yo Intermittent Recovery Test level 1 (Yo-Yo IR1), Maximal Anaerobic Shuttle Running Test (V_MASRT_), Maximal Sprinting Speed Test (20-m flying sprint), Countermovement Jump (CMJ), and Standing Long Jump test (SLJ). The results of the study showed that there were very large negative correlations between tlim100 and SST (sum of sprint times), and large negative correlations between Yo-Yo IR1, Vam-Eval, and SST during RSS in young elite male soccer players (*p* < 0.05). Additionally, V_MASRT_ and SLJ demonstrated a moderate negative correlation with SST (*p* < 0.05). In contrast, significant positive correlations were found between 20-m flying sprint and the SST (*p* < 0.05). According to the stepwise multiple linear regression analysis, the primary predictors of SST, ranked by importance, were tlim100 and Yo-Yo IR1. These two predictors collectively accounted for 72% of the variance in players’ SST (*p* < 0.0001). Due to the importance of aerobic capacity and short repeated accelerations/sprint sets for overall competitive performance in soccer, in conclusion, our results suggest that elite young male soccer players should perform both high intensity interval training and aeorobic capactity exercises as part of soccer training if the primary outcome is to improve repeated sprint ability performance.

## Introduction

It is well-known that, in elite young soccer players, fitness is a multi-component construct involving several physical qualities (e.g., speed, and muscular power) and the activation of both the aerobic and the anaerobic energy systems^1–3^. Therefore, assessing the physical capacities of young soccer players should include composite tests that target several related factors concurrently. Indeed, a number of fitness tests have been used to obtain objective information regarding players’ fitness levels. Laboratory tests are traditionally considered the gold standard^4^, however, in the last years more soccer-specific field performance tests have been designed with the aim to better simulate soccer match play^5^. These tests include jumping assessments to measure muscular power^6^, sprint tests to determine maximal sprinting speed (MSS)^7^, supramaximal intermittent running tests to assess maximal anaerobic speed (MAnS)^8^, and continuous and intermittent tests to determine aerobic fitness characteristics^9^.

Despite the importance of field-based testing providing valid information related to performance in the specific capacity being assessed^10–12^, logistical difficulties may arise in monitoring changes in several physiological variables across a season. Implementing an extensive testing battery at numerous stages during a training year is often problematic owing to scheduled matches, players’ fatigue, and time requirements. Indeed, parts of the scientific and coaching communities have underlined the importance of developing composite tests that provide information related to several physical and physiological requirements in elite young soccer players^1^.

Over the course of the past few decades, the concept of Repeated Sprint Sets (RSS) has emerged, achieving significant recognition within sports research and the applied real-world^13^. RSS pertain to the capacity to recuperate swiftly and replicate performance across multiple sets of sprints^13–15^. Anchored in contemporary research, RSS has gained significance as a pivotal fitness attribute for young soccer players, enabling the study of the activity patterns and physiological responses exhibited by players during a match^5^. Moreover, the assessment of RSS has been considered to offer a more precise and, consequently, authentic depiction of players’ distinct fitness and performance status, thus conferring a heightened level of specificity^16^.

Given the key role that RSS assumes in shaping the performance trajectory of elite young soccer players, strength and conditioning coaches demonstrate a keen interest in optimizing athlete proficiency in RSS^4,13^. To achieve this objective, coaches necessitate a robust comprehension of the determinants underpinning RSS capability. Regrettably, despite a considerable volume of studies investigating the energetic demands associated with a singular set of Repeated Sprint Ability (RSA), there still exists a lack of evidence in the establishment of a cohesive link between conventional fitness parameters and RSA outcomes in male^17–19^ and female soccer players^20^. Furthermore, there is a dearth of published data comprehensively examining the physical and physiological attributes that underpin a novel framework for evaluating the intricacies of multiple sprint sets. Hence, understanding the interplay between RSS performance and field-based tests aimed at assessing specific physiological attributes becomes imperative to ascertain the validity of the RSS test as a comprehensive method for measuring and predicting fitness. Therefore, the aim of the present study was to explore the correlation between RSS proficiency and measurements of maximal sprinting speed, maximal aerobic speed (MAS), MAnS, aerobic capacity, and explosive strength. The primary intent was to ascertain the suitability of the RSS as a tool for evaluating multiple physical attributes and to quantify which field-based tests exhibit the strongest predictive relationship with RSS, particularly within the cohort of elite young male soccer players. In light of this pursuit, it may be hypothesized that an index reflecting aerobic capacity, specifically one that is closely associated with peripheral factors, such as the velocity to exhaustion at 100% of vVO_2_max (tlim100), may potentially exhibit a more robust correlation with RSS than the conventional VO_2_max measurement. Should such an inference prove valid, it would carry notable implications for the formulation of targeted training regimens aimed at augmenting RSS capabilities. Different training modalities have been suggested to optimally enhance either tlim100 or VO_2_max^21–23^, thus underscoring the potential influence of distinct training approaches and specific physical attributes pertinent to soccer performance. The study hypothesis was that a potential correlation between both anaerobic and aerobic performance and the RSS performance in young male elite soccer players would be detected.

## Methods

### Participants

A total of 32 elite male young soccer players, with an average age of 14.6 ± 0.3 years, body mass of 65.5 ± 7.4 kg, height of 170.1 ± 0.1 cm, body fat percentage of 11.7 ± 2.2%, and predicted years from peak height velocity (PHV) of − 0.4 ± 0.3 years participated in this study. The participants had accumulated an average of 3.7 ± 0.5 years of training experience. All participants were in good health, devoid of any significant lower limb injury history, and were not undergoing any medication during the course of the study. The regular weekly training schedule included approximately 14 h of combined soccer-specific training and competition, encompassing six to eight soccer training sessions, one strength training session, one to two conditioning sessions, and one official match. All participants and respective guardians were provided with detailed information regarding the study design, potential risks and benefits, and provided voluntary written informed consent to participate prior to the study commencement. The study was approved by the Local Ethics Committee of the National Centre of Medicine and Science of Sports of Tunis (CNMSSLR09SEP01) prior to the initiation of the assessment procedures and was conducted in compliance with the principles outlined in the Declaration of Helsinki. An a priori sample size calculation was performed using a freely available online tool, G*Power version 3.1.9.7 (www.gpower.hhu.de), with a power level of 80% and an α level of 0.05, based on a previously published study, which reported large to very large/nearly perfect correlations^24^. We took the lowest of these correlations. This analysis revealed that a sample size of > 16 would be sufficient for the analysis.

### Procedures

The experimental procedures were conducted during the last stages of the competitive season, specifically during the months of April and May. The participants were engaged in a total of eight distinct experimental sessions, with 72-h recovery to mitigate the potential impact of fatigue on the subsequent trials. In order to minimize the influence of circadian rhythm variations, all evaluations were consistently administered between the hours of 5:00 p.m. and 8:00 p.m. To ensure familiarity and compliance, players completed a preliminary phase of testing protocol familiarization during the week preceding the commencement of the experiment. All participants conducted a comprehensive battery of physical assessments, including the following: 1) maximal continuous and incremental running test (Vam-Eval) to ascertain MAS, 2) exhaustive constant velocity test conducted at 100% of MAS to determine time to exhaustion (tlim100), 3) 20-m multi-stage shuttle run test (V_MSRT_) to estimate maximal aerobic velocity during MSRT, 4) Yo-Yo Intermittent Recovery Test level 1 (Yo-Yo IR1) to evaluate maximal aerobic power, 5) maximal anaerobic shuttle running test (V_MASRT_) to assess maximal anaerobic speed, 6) MSS Test (a flying 20-m sprint, which specifically involved measuring the time taken to cover the last 10-m of the 30-m sprint effort), 7) vertical and horizontal jump assessments to quantify lower-limb muscular power, and 8) RSS test, comprising two sets of five bouts of 20-m sprints with a 15-s recovery between sprints and a 1-min rest between sets. The parameters necessary for attaining the velocity associated with maximal oxygen uptake (referred to as vVO_2_max) encompass the fulfillment of two specific criteria: voluntary discontinuation due to exhaustion, and the achievement of a heart rate that equals or surpasses 90% of the projected maximum heart rate^25^. All evaluations were completed consistently on a natural grass soccer field, maintaining uniform ambient conditions throughout, with temperatures ranging between 22 and 25°C and relative humidity levels between 40 and 50%. Prior to the test battery, participants completed a standardized warm-up protocol, overseen by both the primary researcher and the coach. Throughout the assessment procedures, players received verbal encouragement and motivation to exert maximal effort.

Participants were categorized into distinct groups based on maturity status and the equation formulated by Mirwald et al.^26,27^. The Maturity Offset, a quantification derived from this equation, was calculated as follows:

$$\begin{aligned} {\text{Maturity}}\;{\text{Offset}} = & - {\text{9}}.{\text{236}} + \left( {0.000{\text{27}}0{\text{8}}*{\text{Leg}}\;{\text{Length}}*{\text{Sitting}}\;{\text{Height}}} \right) + \left( { - 0.00{\text{1663}}*{\text{Age}}*{\text{Leg}}\;{\text{Length}}} \right) \\ & + \left( {0.00{\text{7216}}*{\text{Age}}*{\text{Sitting}}\;{\text{Height}}} \right) + \left( {0.0{\text{2292}}*{\text{Weight}}\;{\text{by}}\;{\text{Height}}\;{\text{Ratio}}} \right). \\ \end{aligned}$$

This method of maturity assessment has been previously applied across a spectrum of studies^28–30^. Notably, it represents a non-invasive and methodologically endorsed approach to anticipate the duration from peak-height-velocity (PHV), thereby serving as a surrogate measure of maturity offset through the utilization of pertinent anthropometric variables.

### Data collections tools

#### Anthropometric measures assessment

The players’ height was ascertained using a wall-mounted stadiometer, while body mass was measured employing an electronic scale (Scale Electronics Development, New York, USA). The sum of skinfolds (biceps, triceps, subscapular, and suprailiac) was monitored with a Harpenden skinfold callipers (Baty International, West Sussex, United Kingdom). Body composition measurements were conducted according to the procedure of Deurenberg et al.^31^ who reported similar prediction errors between adults and young adolescents. The measurements were meticulously executed by a single proficient investigator with ISAK 2 certification, ensuring consistency and accuracy in the assessment process.

#### Repeated-Sprint sets (RSS)

The protocol consisted of two sets of 5 × 20-m sprints with a 15-s recovery between sprints and 1-min between sets^13,16^. At the end of each sprint, the players engaged in a 10-m deceleration phase, followed by a 10-m active jog recovery period. Sprint times were recorded using Electronic Timing gates (Brower Timing System, Salt Lake City, UT, USA). This system has been previously assessed for validity and reliability with reported accuracy of 0.01-s^13,32^. The recorded performance measure encompassed the sum of sprint times achieved during the two sets (SST). The Interclass Correlation Coefficient (ICC) and the Coefficient of Variance (CV) for RSS test were 0.93 and 0.37% respectively for SST^16^. The heart rate (HR) was continuously recorded during the RSS using a Heart Rate Monitor (Polar Accurex Plus, Kempele, Finland). Moreover, finger-tip capillary lactate concentrations ([Lac]) were measured before and 3-min following the RSS test using a hand-held Lactate Pro device (Arkray, KDK, Japan). Following the RSS test, the participants’ rating of perceived exertion (on a scale of 0–10) was recorded^33^.

#### VAM-EVAL track test (Vam-Eval)

To estimate MAS a modified version of the University of Montreal Track Test^34^, known as Vam-Eval, was employed. The test began with an initial running speed of 8.5-km h^−1^ with consecutive speed increases of 0.5-km h^−1^ each minute until exhaustion. The players adjusted running speed according to auditory signals timed to match 20-m intervals delineated by marker cones around a 200-m long indoor athletics track. The test ended when players failed on two consecutive occasions to reach the next cone in the required time. The average velocity of the last stage completed was recorded as the players’ vVO_2_max (km h^−1^). The typical error, expressed as a CV, was calculated to be 3.5%, with 90% confidence limits ranging from 3.0 to 4.1%^35^.

#### 20-m multi-stage shuttle run test (V_MSRT_)

The MSRT was conducted as previously described by Léger et al.^36^. This test consisted of shuttle running between two lines spaced 20-m apart. The initial velocity of the incremental test was set at 8.5-km h^−1^ and was increased by 0.5-km h^−1^ every minute. The participants adjusted running velocity according to a combination of regular auditory pacing signals provided by a calibrated beeper (Best Electronic, France). The test ceased when: (1) the participant could no longer sustain the running pace or (2) the participant failed to arrive within 2-m of the end line on two consecutive occasions. The estimated velocity associated with VO_2_max was calculated as the speed of the last fully completed stage. The test–retest reliability coefficient has previously been reported to be 0.89, indicating a high level of consistency and reliability^36^.

#### Yo-Yo intermittent recovery test level 1 (Yo-Yo IR1)

This test involves running back and forth between two lines 20-m apart, aiming to cover the maximum distance possible. During the test, participants receive a 10-s rest between every 40-m run. The running pace is incremental and set by an auditory beep. The participants must stand stationary behind the 20-m line at the same time as the beep sounds. The test ends when the participant cannot continue due to fatigue or when the participant fails to arrive on the end line before the beep sounds on two consecutive occasions. During this test, the participant’s performance is assessed based on the total distance covered, regardless of whether or not the full stage was completed^37^. The Yo-Yo IR1 reliability has previously been established with an ICC of 0.98 and a CV of 3.5%. These values indicate a high level of reliability and consistency^38^.

#### Time to exhaustion at 100% of MAS (tlim100)

A constant velocity test to exhaustion (tlim100) was conducted on the same 200-m track, using 20-m reference marks. The test involved running at a velocity corresponding to 100% of vVO_2_max (velocity at VO_2_max) until the point of exhaustion. Following a 15-min warm-up at 60% of individual vVO_2_max, participants were instructed to run for as long as possible at individual vVO_2_max. During the test, the assessor used sound signals to indicate the desired speed required. This was facilitated by a stopwatch that had adjustable countdowns, allowing precise timing to the nearest 1/100th of a second to be set. The countdowns and accompanying sound signals provided participants with information to maintain the specified speed by reaching the designated marks corresponding to each signal. The inter-session reliability of the time to exhaustion at vVO_2_max test in children and adolescents showed a CV value of 22%^39^.

#### Maximal anaerobic shuttle running test (V_MASRT_)

The V_MASRT_ consisted of 20-s shuttle runs with a 100-s passive recovery period between the runs. This test consisted of intermittent shuttle running between two lines spaced 20-m apart. The initial velocity of the first stage was set at 100% of the individual’s maximal aerobic velocity recorded from the 20-m MSRT. The velocity was increased by 0.28-m s^−1^ for each consecutive stage of 20-s shuttle runs until volitional exhaustion. The participants had to run for 20-s from the start line to the opposite line, touch the line with a self-selected foot, turn with a 180° change of direction, and run back to the start line within the designated audio beeps. The computer software program also organizes time of effort (20-s) and recovery (100-s) as previously described^40^. The average velocity of the last 20-s shuttle run completed was recorded as the individual V_MASRT_ (km/h). The ICC for test–retest reliability of the V_MASRT_ test has been previously reported to be 0.4^40^. The typical error of measurement for the V_MASRT_ test was calculated as 3.6%, which represents the expected amount of variability or random error in the measurements^40^.

#### Maximal sprinting speed (MSS)

The MSS was determined using a 30-m sprint effort with electronic timing gates (Brower timing system, Salt Lake City, UT, USA; accuracy of 0.01^−1^ s) with split times at 10-m, and 30-m. The participants were instructed to run as quickly as possible along the 30-m distance from a standing start. The MSS was evaluated by conducting a flying 20-m sprint, which specifically involved measuring the time taken to cover the last 10-m of the 30-m sprint effort^40^. Speed was measured to the nearest 0.01^−1^ s. The ICC for a flying 20-m sprint has been reported to be 0.90. This ICC value indicates a strong level of agreement and reliability between repeated measurements of the flying 20-m sprint^3,5^. The participants completed two trials of the sprint, with a minimum of 3-min rest between each trial. The best performance of the two trials was used for analysis.

#### Countermovement jump (CMJ)

Participants initiated the test from an upright standing position and executed a rapid downward eccentric action, followed immediately by a vertical jump for maximal height. During the entire test, players were instructed to maintain hand positioned at the hips to prevent any involvement or influence from arm swing^41^. Three trials were conducted, with approximately two minutes of recovery between each trial. The best result achieved among the three trials was selected for further analysis. The ICC for the CMJ was calculated to be 0.97, with a 95% CI ranging from 0.93 to 0.99^42^.

#### Standing long jump (SLJ)

Prior to the test, participants stood stationary with toes aligned with the start line and were instructed to push off aggressively and jump forward as far as possible, aiming to cover as much distance as possible. During the jump, the players were allowed to utilize a countermovement, involving arm and body swing, to generate additional power and enhance the distance achieved in the jump. The distance covered by the jump was measured from the start line at take-off to the point where the back of the heel made contact with the ground, nearest to the take-off line. The measurement was conducted using a metal tape measure and recorded in centimeters. The test was repeated three times, and the maximum distance achieved was recorded and used for analysis^43^.

### Statistical analysis

The data were analyzed using the commercial software Statistical Package for Social Sciences (SPSS), version 17.0 (SPSS Inc., Chicago, IL, USA). All experimental data are presented as means ± standard deviation (SD) with a 95% confidence interval (95% CI). Prior to conducting statistical analyses, the normal distribution of the variables was assessed using the Kolmogorov–Smirnov test. Pearson product moment correlations were performed to ascertain the correlation between each performance variable and the SST performance. The magnitude of correlation between tests measures were assessed with the following thresholds: < 0.1, trivial; < 0.1–0.3, small; < 0.3–0.5, moderate; < 0.5–0.7, large; < 0.7–0.9, very large; and < 0.9–1.0, almost perfect^44^. In cases where the CI of a variable overlapped with small positive and negative values, the magnitude of the effect or difference was considered unclear. However, if CI did not overlap and displayed a clear separation from zero, the magnitude was deemed to be the observed magnitude of the effect or difference^44^. Separate linear regression models were fitted to establish the relationship between each fitness test (dependent variable) and SST (independent variable). Finally, Multiple Linear Regression Models (stepwise backward elimination procedure) with SST as the dependent variables were also used. Statistical significance was set at *p* ≤ 0.05.

### Ethics approval and consent to participate

The study was reviewed and approved by the National Centre of Medicine and Science of Sports, Tunis (CNMSS-LR09SEP01). Written informed consent to participate in this study was provided by the participant’s legal guardian/next of kin. Informed consent was obtained from all individual participants included in the study.

## Results

**Figure 1:**
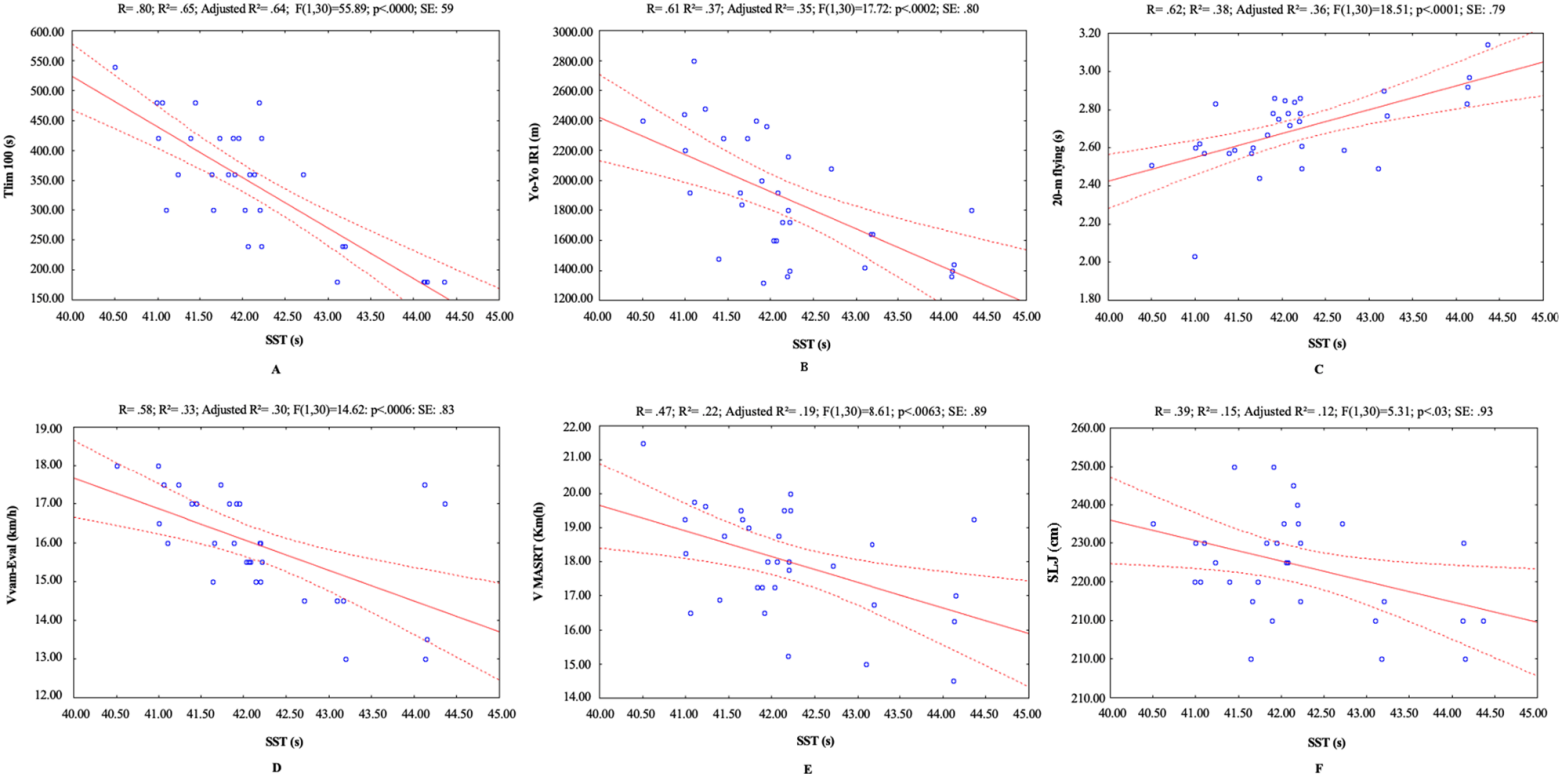
Relationships (90% confidence intervals) between different variables of Sum of Sprint Times. *Note* (**A**) Correlation between tlim100 and SST; (**B**) Correlation between Yo-Yo IR1 and SST; (**C**) Correlation between 20-m flying sprint and SST; (**D**) Correlation between V_Vam-Eval_ and SST; (**E**) Correlation between V_MASRT_ and SST; (**F**) Correlation between SLJ and SST.

The results for RSS are shown in Table 1. The mean values and SD of the diverse assessed variables are presented in Table 2. With the exception of the CMJ performance, the associations between each dependent variable and the SST performance are graphically depicted in Fig. 1.

**Table 1:** Descriptive statistics (all values presented as means ± SD) of Repeated Sprint Sets.

Variables	Mean ± SD	95% CI
SST (s)	42.1 ± 0.9	41.85–42.52
MHR (bpm)	183.7 ± 8.1	180.82–186.73
Post-test blood lactate (mmol·L^−1^)	5.7 ± 1.1	5.35–6.19
RPE (total score)	7.7 ± 1.34	7.26–8.23

*SST* Sum of Sprint Times, *MHR* maximal heart rate, *RPE* rate of perceived exertion.

**Table 2:** Descriptive statistics (all values presented as means ± SD) of physical performance.

Variables	Mean ± SD	95% CI
SLJ (cm)	224.8 ± 13.5	219.84–228.90
CMJ (cm)	33.6 ± 3.1	32.55–34.73
MSS 20-m flying sprint (s)	2.7 ± 0.2	2.62–2.75
V_MASRT_ (km/h)	18.0 ± 1.5	17.45–18.56
V_MSRT_ (km/h)	13.4 ± 0.8	13.11–13.64
V_Vam-Eval_ (km/h)	15.1 ± 1.3	15.48–16.42
tlim100 (s)	339.3 ± 104.4	303.79–373.12
Yo-Yo IR1 (m)	1880.6 ± 404.4	1741.26–2019.34

*CMJ* Countermovement Jump, *SLJ* Standing Long Jump, *MSRT* Multi-Stage Shuttle Run Test, *SST* Sum of Sprint Times, *tlim100* a constant velocity test performed until exhaustion at 100% of vVO_2_max, *MASRT* Maximal Anaerobic Shuttle Running, *MSRT* Multi-Stage Shuttle Run Test, *MSS* Maximal Sprinting Speed, *Yo-Yo IR1* Yo-Yo Intermittent Recovery Test Level 1.

A very large significant and strong negative correlation (r = − 0.80, 95% CI [− 0.90 to − 0.63], *p* = 0.07) was observed between the tlim100 and the performance in the SST. Notably large negative correlations were evident between the Yo-Yo IR1 performance (r = − 0.61, 95% CI [− 0.79 to − 0.33]) as well as the V_Vam-Eval_ performance (r = − 0.57, 95% CI [− 0.76 to − 0.28]) and the SST performance. Similarly, large positive correlations (r = 0.61, 95% CI [0.35–0.79]) were identified between the 20-m flying sprint performance and the SST. Both the V_MASRT_ performance (r = − 0.47, 95% CI [− 0.70 to − 0.14]) and the SLJ performance (r = − 0.38, 95% CI [− 0.64 to − 0.04]) displayed moderate yet negative correlations with the SST performance. Notably, no statistically significant correlations were observed between the CMJ performance and the SST performance (r = − 0.22; *p* > 0.05).

The outcomes of the Linear Regression Analysis unveiled noteworthy predictive relationships. Specifically, the performance in the SST demonstrated significant predictability, accounting for 65%, 37%, 38%, 33%, 22%, and 15% of the variance in the time to exhaustion at 100% of maximal aerobic speed (tlim100), the 20-m flying sprint performance, the Yo-Yo IR1 performance, V_Vam-Eval_ performance, V_MASRT_ performance, and the SLJ performance, respectively (Fig. 1).

Moreover, employing Stepwise Multiple Linear Regression Analysis, the foremost predictors contributing to SST performance were identified in a hierarchical order. Notably, the most influential predictors were found to be tlim100 and Yo-Yo IR1. Together, this dual-predictor model elucidated a substantial proportion of the variance in players’ SST performance, collectively accounting for 72% of the observed variability (R^2^ = 0.72, *p* < 0.0001) (Table 3).

**Table 3:** Results of Stepwise Multiple Regression Analysis of predictors that influenced SST performance.

Model	Unstandardized coefficients		Coefficients beta	Sig.	R^2^
	B	Std. error			
SST					
(Constant)	6.05	2.74	–	0.002	
Yo-Yo IR1	0.03	0.01	0.50	0.001	0.20
tlim100	3.91	1.07	0.72	0.001	0.32
MSS 20-m flying sprint	1.04	0.41	0.17	0.78	0.10
V_Vam-Eval_	1.78	0.55	0.19	0.86	0.11
V_MASRT_	2.11	1.12	0.14	0.97	0.12
SLJ	1.96	1.03	0.12	0.76	0.15

*SST* Sum of Sprint Times, *Yo-Yo IR1* Yo-Yo Intermittent Recovery Test Level 1, *tlim100* a constant velocity test performed until exhaustion at 100% of vVO_2_max, *MSS* Maximal Sprinting Speed, *SLJ* Standing Long Jump, *MASRT* Maximal Anaerobic Shuttle Running.

## Discussion

The data from the present study reveal that there is a strong correlation between RSS and tlim100, MAS, and MSS. However, the correlation between performance in repeated-sprint sets (SST performance) and tests such as SLJ, V_MASRT_ (15% and 22%, respectively) was found to be trivial, indicating that these variables may not be strongly related. Lastly, Stepwise multiple linear regression analyses were conducted to explore the major predictors of RSS. The results of these analyses revealed that the two most important predictors of RSS, in order of importance, were time to exhaustion at 100% of vVO_2_max (tlim100) and Yo-Yo IR1. According to the present results, composite tests that provide information related to several physiological capacities are of considerable interest for conditioning coaches in the real-world.

The present investigation has substantiated a significant correlation between the relative RSS and tlim100, accounting for 65% of the shared variance. Consistent with our findings, numerous researchers have also evidenced strong and statistically significant associations between aerobic capacity and repeated sprint performance^45–48^. This alignment with our outcomes can potentially be attributed to the supposition that repeated sprint performance places heightened emphasis on localized adaptations concerning the replenishment of phosphocreatine (PCr) and the capacity to generate force. This may offer an explanatory basis for the pronounced correlation between repeated sprint performance and aerobic capacity as evidenced in our study. Worth noting is that the tlim100 protocol has been acknowledged as an indirect surrogate for peripheral aerobic fitness^18,49^. This supposition is further fortified by the work of Da Silva et al.^45^, who identified a more potent correlation between the velocity associated with the onset of blood lactic acid (OBLA) and Repeated Sprint Ability (RSA) than with VO_2_max. The onset of blood lactic acid is considered to more accurately reflect peripheral adaptations resultant from aerobic training, and is predominantly shaped by biochemical alterations within the muscle fibers^50,51^. Additionally, substantial correlations have been documented between RSA and indices such as muscle oxidative capacity, lactate removal efficiency^19,40,52^, and the lactate threshold^46,53,54^. The interrelation between these parameters is complex, accentuated by the observations of Psotta et al.^48^, who noted significant correlations (r = 0.62, *p* = 0.01) between the average speed during repeated sprint tests and the speed corresponding to the ventilatory threshold. The empirical data derived from the current study thus lends credence to the proposition that aerobic capacity and RSS potentially share an underlying metabolic or energetic constituent.

Our findings are in concordance with the strong correlations observed between repeated-sprint performance and MAS in young soccer players as documented in prior studies^9,45,55,56^. Moreover, the present results serve to corroborate the fundamental role of maximal aerobic power, predominantly governed by central factors, particularly in the context of metabolic recovery amid successive sprints^40,51^. It is pertinent to note that the capacity to engage in repeated sprints is profoundly contingent upon the restitution of PCr following each sprint^7^, a process tied to the availability of oxygen^57^. While the relative prominence of MAS in the context of RSA can be influenced by specific testing protocols, the current dataset underscores how modifications in aerobically-associated locomotor performance and indirect muscle oxidative capacity^56^ can exert a discernible impact on repeated-sprint sequences.

The lactate concentration measured following the RSS test was found to be 5.7 ± 1.1 mmol l^−1^. This result is in agreement with Selmi et al.^13^, who reported lactate concentrations of 6.45 ± 1.97 with junior (U17) soccer players employing a similar RSS protocol to the present study. This increase in lactate may be due to the fact that the recovery period was too short for a complete resynthesis of PCr. In fact, it has been reported that short intervals of recovery between sprints (≤ 30-s) will result in a progressive decrease of the muscle PCr stores, therefore placing a higher demand on anaerobic glycolysis for the regeneration of Adenosine Triphosphate (ATP)^50,58,59^. This low lactate concentration suggests moderate solicitation of anaerobic glycolysis during the RSS test, and was confirmed by the significant relationship reported in the present study between RSS and V_MASRT_, explaining 22% of the shared variance. These results may be somewhat surprising, as both types of tests, the RSS test and V_MASRT_, are intermittent running exercises involving the same effort-rest ratio of 1:5. Moreover, one possible reason for the negative significant correlation between V_MASRT_ and RSS test may derive from the contribution of aerobic metabolism during the V_MASRT_ test, which is supported by Garbi et al.^60^, who reported a significant correlation between V_MASRT_ and VO_2_max (r = 0.63, *p* < 0.01). Additionally, Zagatto et al.^61^ found that, during the maximal anaerobic running test, aerobic and anerobic energy system contributions were 65.4% and 34.6%, respectively. V_MASRT_ has a 100-s rest period after each effort, which leads to further aerobic contribution to increase lactic acid removal and PCr restoration for recovery, thus helping maintain performance across multiple sprint sets.

Furthermore, our collected data underpins the significant role of MSS as a substantial contributor to the RSS performance, with 38% of the variance in RSS outcomes being explicated by the results of the 20-m flying sprint performance. The present findings are in line with the observations made by Pyne et al.^62^, who documented a moderate correlation (r = 0.66) between a repeated-sprint test encompassing six sets of 30-m sprints and the time taken for a 20-m sprint in junior male soccer players. Moreover, Mendez-Villanueva et al.^63^ demonstrated a similar moderate correlation between a repeated-sprint test (consisting of 10 sets of 30-m sprints) and MSS represented by the flying 20-m sprint time across three distinct junior soccer player groups (U14, U16, and U18). Indeed, the stronger correlation observed with a metric assessing MSS, specifically the flying 20-m sprint, is in line with prior reports that emphasize the proximity of RSS to the functioning of the alactic energy system, encompassing processes such as PCr degradation within muscles^64^, and the muscle’s buffer capacity^65^.

Conversely, in the current study, a significant correlation between horizontal jumping abilities and the RSS was detected, representing 15% of the shared variance. This phenomenon may be partially due to the specific form of jumps administered, namely the SLJ, although it is acknowledged that the contribution of the stretch–shortening-cycle (SSC) to the initial phase of a sprint or jump is deemed to be minimal^66,67^.

Possibly, utilizing exclusively concentric jumps may therefore yield enhanced predictive capabilities compared to the employment of SSC jumps as employed within the present study. Practitioners manifest a notable interest in discerning the correlations among diverse fitness attributes in relation to the variables of age and maturation. This pursuit is aimed at the establishment of coherent and enduring training protocols intended for the progressive cultivation of emerging soccer talents^68^.

The present study, while contributing valuable insights, does suffer from certain limitations that warrant consideration. Foremost, our investigation focused exclusively on a cohort of youth male soccer players, thus the findings are constrained to this particular demographic and generalizations must be considered with caution. Moreover, it is imperative for future research to encompass female and male cohorts spanning diverse training regimens and stages of physiological maturation, potentially incorporating innovative methodologies for administering RSS interventions. Furthermore, a control group was not incorporated within the present study design and may be beneficial in future research. It may also be worthwhile to consider examining RSS training programs and the effects on varying positions. Thus future research exploring specific training strategies that may maximise the potential relationships between RSS and physiological parameters to enhance player performance is warranted.

## Conclusion

The findings of the current study revealed that the foremost predictors influencing the outcomes of the SST, ranked by significance, are tlim100 and Yo-Yo IR1. Remarkably, the combined influence of these two predictors accounts for a substantial 72% of the observed variance in SST performance among elite youth male soccer players. Considering the direct correlation between aerobic capacity and the ability to engage in repeated sprint performances, both of which are pivotal elements contributing to success in competitive soccer, it becomes imperative to transfer the outcomes of this study into youth soccer training programs. Coaches are strongly advised to be aware of the insights gleaned from the present study while working with young soccer players, diligently incorporating both aerobic and RSS training modalities. Consequently, for those aiming to enhance RSA and improve aerobic capacity within the context of youth soccer, the strategic integration of RSS training emerges as a key and pivotal pedagogical directive.

The participants in the present study were predominantly skilled youth male soccer players with a substantial level of experience. To ascertain a comprehensive understanding, it is advisable to compare the outcomes of this study with those obtained from novice players. It is reasonable to ascertain that RSS exercises may exhibit greater suitability for both less-experienced and more experienced soccer players. Future research should examine the mechanisms underpinning the observed correlations and explore alternative testing methodologies that may complement RSS in assessing player performance.

## Data Availability

Data are available for research purposes upon reasonable request to the corresponding author.
